# Six-minute stepper test in hospitalized elderly patients: Convergent validity, test-retest reliability and safety

**DOI:** 10.1371/journal.pone.0241372

**Published:** 2020-10-29

**Authors:** Davi de Souza Francisco, Larissa Martinez, Aline Carleto Terrazas, Diego Britto Ribeiro, Wellington Pereira Yamaguti

**Affiliations:** Hospital Sírio-Libanês, Rehabilitation Service, São Paulo, Brazil; University of Rouen-Normandie, FRANCE

## Abstract

**Objective:**

To evaluate the convergent validity of the six-minute stepper test (6MST) with the variables used in the diagnosis of sarcopenia (appendicular muscle mass, handgrip strength and six-meter gait speed test), as well as to evaluate test-retest reliability and safety when applied to hospitalized elderly patients. Finally, we aimed to compare the performance in the 6MST between hospitalized elderly patients and healthy elderly from the community.

**Materials and methods:**

Observational and cross-sectional study. Elderly patients admitted to a private hospital and healthy elderly from the community were recruited. On the first day, the patients included underwent the following assessments: anthropometric, handgrip strength (HGS), six-meter gait speed test (6GST) and 6MST. On the second day, before breakfast, patients underwent body composition assessment. The healthy elderly were evaluated on a single day and performed only anthropometric assessment and 6MST.

**Results:**

30 hospitalized patients (age 71.0±7.9 years) and 15 healthy elderly (age 68.1±5.8 years) were included. There was a high correlation of 6MST with 6GST (r = 0.78; p<0.001), moderate correlation with HGS (r = 0.59; p<0.001) and low correlation with appendicular muscle mass (r = 0.45; p = 0.01). There was no statistical difference between the first and second 6MST performed by hospitalized elderly (196.2±91.0 cycles vs. 191.3±103.7 cycles; p = 0.66), in addition to an excellent agreement between these measures (ICC = 0.90; 95% IC 0.78–0.95). Only one adverse event (3.3%) occurred in the sample.

**Conclusion:**

6MST showed convergent validity with the functional variables used in the diagnosis of sarcopenia. In addition, excellent test-retest reliability was observed, which indicates the need for a single assessment in hospitalized elderly patients. The prevalence of adverse events during the application of the test is low, without resulting in clinical symptoms; therefore, the test is considered safe for this population. In addition, hospitalized elderly patients perform worse in the 6MST compared to healthy elderly from the community.

## Introduction

The aging process generates structural changes in the various body systems, contributing to functional worsening [1–3]. An example of this is sarcopenia, a condition with a higher prevalence in the elderly, characterized by reduced muscle function and that favors exercise intolerance [4–6]. In addition, the presence of chronic diseases and the need for hospitalization are factors that can potentiate this decline [7–10]. Thus, it is important to use functional tests to assess hospitalized elderly, since studies have already shown a strong association between reduced exercise capacity and mortality [11–13].

The six-minute walk test is considered the gold standard for submaximal assessment of exercise tolerance, being widely used in the scientific field. However, this tool has limitations that prevent its applicability in a hospital environment, such as the need for a 30-meter corridor with low circulation of people [14]. In this context, other functional tests, such as the six-minute stepper test (6MST), began to be studied in recent years, aiming to facilitate the assessment of exercise capacity in a clinical environment [15–18].

The 6MST is a simple tool that favors the assessment of patients in isolation, at the bedside and with the maintenance of continuous monitoring. In addition, it has already proved to be a valid, reproducible and reliable instrument for measuring exercise tolerance in the elderly and patients with chronic obstructive pulmonary disease [15, 16, 19, 20]. However, no study to date has investigated the clinimetric properties of the 6MST when applied to a hospitalized population. This draws attention, because several factors related to hospitalization could affect performance in the field test, and a high floor effect would compromise the property of discriminating patients with low and high tolerance to exercise. It could potentially affect the validity of the test in this context. Therefore, the objective of this study was to evaluate the convergent validity of the 6MST with the variables used in the diagnosis of sarcopenia (appendicular muscle mass, handgrip strength and six-meter gait speed test), as well as to evaluate the test-retest reliability and safety when applied to hospitalized elderly patients. Finally, we aimed to compare the performance in 6MST between hospitalized elderly and healthy elderly in the community.

## Materials and methods

### Ethics

It is characterized as an observational, cross-sectional study that was approved by the Human Research Ethics Committee of Sírio-Libanês Hospital (HSL 2019–15; CAAE: 08285119.2.0000.5461). All participants signed an informed consent form.

### Participants

The sampling of study was of the non-probability method, for convenience, recruiting elderly patients admitted to a private hospital in São Paulo—Brazil, and recruiting healthy elderly from the community. The following inclusion criteria in the study were used for hospitalized elderly: (1) age ≥ 60 years; (2) presenting clinical stability (ventilatory and hemodynamic); (3) being between the 3rd and 10th hospitalization days; (4) not having metallic implants; (5) not having neurological and osteomioarticular diseases that would limit the performance of the evaluations; (6) ability to walk independently or with auxiliary devices; and (7) presenting an adequate score on the Mini Mental State Examination, according to the level of education. For the healthy elderly from the community, the following inclusion criteria were considered: (1) age ≥ 60 years; (2) not having cardiac, pulmonary, metabolic, neurological or osteomioarticular disease; and (3) not having undergone recent surgery (<30 days for medium and <60 days for large). The exclusion criteria for all participants were: (1) inability to perform the evaluations within the criteria of technical acceptability and (2) cardiorespiratory instability during the tests (angina, elevation of the heart rate above 80% of the maximal theorical heart rate and oxygen pulse saturation below 88%).

### Measurements

#### Charlson comorbidity index (CCI)

The CCI was used to classify the presence of comorbidities. The patient’s score depends on the amount of comorbidities [21, 22].

#### Mini mental state examination (MMSE)

The cognitive level assessment was carried out by means of the MMSE. The version by Brucki et al. (2003) [23] was used, and we considered the cutoff points ≥ 20 points for illiterate individuals and ≥ 24 for schooled individuals [24].

#### Anthropometric assessment

For anthropometric evaluation, a stadiometer (Filizola, Personal) was used to measure height. The participants were instructed to remove their shoes, stand upright and keep their head aligned. The body mass index (BMI) was calculated after the measurement of body mass by the bioimpedance monitor. Participants were classified by BMI as skinny (<18.5 kg/m^2^), eutrophic (18.5–24.99 kg/m^2^), overweight (25–29.99 kg/m^2^), and obese (≥ 30 kg/m^2^) [25].

#### Body composition assessment

The body composition was assessed by electrical bioimpedance, using a segmental body composition monitor (BC-558 Ironman; Tanita). This device emits an electrical current of 50 kHz through four electrodes on the platform that is in contact with the feet and four more electrodes that are in contact with the hands. To assess the patients, they were instructed to climb on the monitor without shoes, without ornaments and keep their feet and hands in contact with the electrodes, until the end of the measurement [26]. The evaluations were carried out before breakfast, according to the recommendations of Kyle et al. (2004) [27]. The measured and used variables for analysis were: body mass (kg), percentage of body fat tissue (%), total muscle mass (kg) and appendicular muscle mass (kg), which is a total muscle mass of each member of the patient.

#### Peripheral muscle strength assessment

The patient’s peripheral muscle strength was assessed by the handgrip strength test (HGS) of the dominant upper limb. A manual hydraulic dynamometer (SH 5001, SAEHAN) was used and the recommendations of the American Society of Hand Therapists [28] were followed. The patient was positioned sitting on a chair, with the non-dominant hand resting on the thigh. Regarding the evaluated limb, the patient was instructed to keep the shoulder in a neutral position, elbow in 90° flexion and the forearm in neutral rotation. Three measurements were taken, considering a difference lower than or equal to 10%. The highest value was considered for analysis. During measurements, the evaluator issued a standardized verbal stimulus and performed a one-minute rest period between each measurement.

#### Six-meter gait speed test (6GST)

The 6GST was used to assess physical performance. The patient was instructed to walk a distance of 10 meters at the highest possible speed, without running. The points were marked: zero, two, eight and 10 meters, to assist in time control. The timer was started when a patient’s lower limb crossed the two-meter mark and was stopped when a lower limb reached the eight-meter mark. Thus, the time to cover the initial and final two meters, which are periods of acceleration and deceleration, was disregarded [29]. Three measurements were taken, with an interval of one-minute rest between them, considering a difference lower than or equal to 10%. The highest value was considered for analysis.

#### Six-minute stepper test (6MST)

The evaluation of the exercise capacity was performed in an isolated room, using a stepper (Mini Stepper Comfort, Domyos). The same recommendations and standardized incentive phrases of the American Thoracic Society and the European Respiratory Society for the six-minute walk test were used for the 6MST [14]. The participant was instructed to support the upper limbs on the support rod of the device only in case of imbalance or exhaustion and to perform as many cycles as possible in the six-minute period, the cycle being defined as the return to the initial position [15, 20]. Before the test was performed, the participants were instructed to position themselves for two minutes with the right or left foot, according to their choice, in the elevated position (20 cm height) and the other foot in the lower position, in order to become familiarized with the device. Parameters such as heart rate (HR), oxygen pulse saturation (SpO_2_), and subjective sensation of dyspnea and fatigue of the lower limbs (assessed using the modified Borg scale) were measured at rest, in the second and fourth minutes of 6MST and immediately after the end of the test. Blood pressure (BP) was measured only at the beginning and immediately after 6MST. To estimate mean arterial pressure (MAP), systolic blood pressure (SBP) and diastolic blood pressure (DBP) were used, in the equation: MAP = SBP + (2 x DBP) / 3. All parameters were reassessed in the period of recovery, six minutes after the end of the test. Two tests were performed, with an interval of 30 minutes between them for rest and return of vital signs, mentioned above, to baseline levels. The test with the best performance was considered for analysis.

#### 6MST safety assessment

To assess the safety of 6MST as a tool for measuring exercise capacity in hospitalized elderly, the prevalence of the following adverse events was considered: fall, syncope, angina, hypotension (MAP < 65mmHg), hypertension (MAP > 120mmHg), HR > 80% of maximal theorical HR, HR < 50bpm, SpO_2_ < 88%, loss of nasoenteral tube and loss of venous access. In addition, the return of vital signs values and the subjective sensation of dyspnea and fatigue of lower limbs to the baseline level were considered in the recovery period after test.

### Design of study

**Fig 1:**
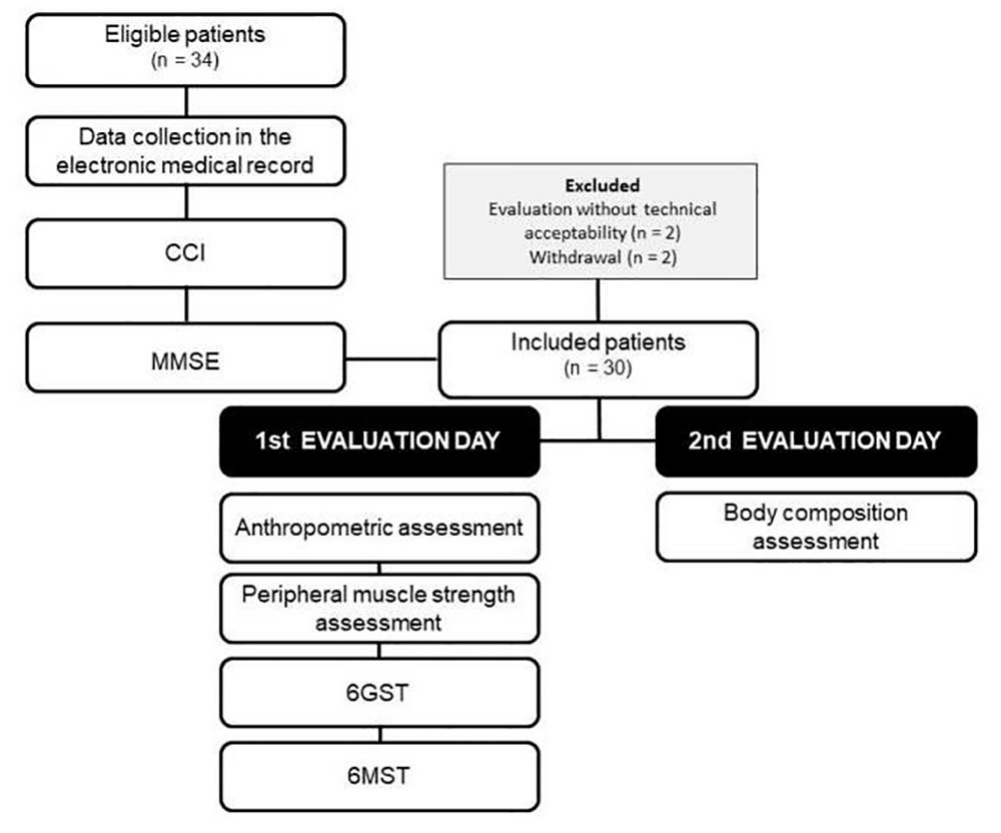
Flowchart of data collection. CCI: Charlson comorbidity index; MMSE: mini mental state examination; 6GST: six-meter gait speed test; 6MST: six-minute stepper test.

The evaluations of hospitalized elderly patients occurred on two consecutive days. On the first day, patients eligible for the study were screened using the institution’s electronic medical record. In addition, a form was filled out with demographic, socioeconomic, clinical and hospitalization data. The CCI was completed. Subsequently, the participants were informed about the research and carried out the assessment of the cognitive level, by means of the MMSE, to be included in or excluded from the study. On the first day, the included patients also performed the following procedures: anthropometric assessment; assessment of peripheral muscle strength; 6GST; and 6MST. On the second day, before breakfast, patients were assessed for body composition, using bioimpedance (Fig 1). The usual assistance with motor physiotherapy was not performed during the period in which the patients were evaluated. The healthy elderly from the community were evaluated on a single day and performed only anthropometric assessment and 6MST.

### Statistical analysis

The sample size calculation was performed considering a correlation coefficient of 0.49, a test power of 80% and an alpha of 0.05 between the gait speed (variable used in the diagnosis of sarcopenia), and the performance in an exercise capacity test [30]. Thus, the inclusion of 30 patients in the study was estimated. Data analysis was performed using the statistical program GraphPad Prism 8 and the level of significance adopted was p<0.05. The Shapiro-Wilk test was used to assess the normality of the data, and they are presented as mean and standard deviation or as median and interquartile range, according to their distribution on the Gauss curve. Pearson’s correlation coefficient was used to correlate 6MST with appendicular muscle mass, HGS, and 6GST in hospitalized elderly patients. The magnitudes of the correlations were considered, such as: 0.26 to 0.49 low; 0.50 to 0.69 moderate; 0.70 to 0.89 high; and 0.90 to 1.00 very high [31]. To analyze the test-retest reliability of the 6MST in a hospital environment, a comparison was made between the first and the second 6MST, using the paired t-test, as well as an analysis of the intraclass correlation coefficient (ICC). ICC < 0.4 was considered low, 0.4 < ICC < 0.75 as good, and ICC > 0.75 as excellent [32]. The Bland-Altman plot was performed to demonstrate the agreement between these measures. To compare vital signs and subjective sensation of dyspnea and fatigue of lower limbs at rest, at the end of 6MST and after recovery, ANOVA one-way tests for repeated measures or Friedman were used. To compare data between elderly male and female patients, as well as to compare data between hospitalized and healthy elderly people, independent t-tests for parametric variables and Mann-Whitney U tests for non-parametric variables were used.

## Results

Thirty hospitalized elderly patients (50% male), with a mean age of 71 years, were evaluated. The median indicated that most patients were evaluated on the fifth day of hospitalization. The most frequent comorbidities were: hypertension (46.7%) and cancer (43.3%). The main causes of hospitalization were for clinical reasons (66.7%), followed by for surgical reasons (33.3%). It was observed that patients performed an average of 211 cycles in the 6MST. The difference of the mean of the lowest values for HGS (24.3 kgf) and 6GST (1.3 m/s) with the mean of the highest values (26.7 kgf and 1.4 m/s, respectively), did not exceed 10%. There was a statistical difference between the male and female genders in the variables: weight, height, total muscle mass, appendicular muscle mass, percentage of body fat and HGS (Table 1).

**Table 1:** Characterization of the sample.

Characteristics	Sample (n = 30)	Men (n = 15)	Women (n = 15)	p
**Age (years)**	71.0±7.9	71 (67–77)	66 (62–77)	0.18
**Weight (kg)**	71.8±14.4	78.5±12.7	65.1±13.1	0.008*
**Height (m)**	1.7±0.1	1.7±0.1	1.6±0.1	<0.001*
**BMI (kg/m**^**2**^**)**	26.2±4.8	25.1 (23.2–28.2)	25.7 (20.5–28.9)	0.93
**Evaluation day (day)**	5 (4–6)	5 (3–8)	5 (4–6)	0.73
**MMSE (points)**	29 (27–29)	29 (28–29)	29 (27–29)	0.50
**CCI (points)**	1 (0–3)	1 (0–3)	1 (0–3)	0.90
**Total muscle mass (kg)**	47.0±9.4	53.6 (50.1–57.3)	41.4 (34.9–44.0)	<0.001*
**Appendicular muscle mass (kg)**	20.2±4.2	23.0 (20.9–24.9)	17.6 (14.6–19.6)	<0.001*
**Body fat tissue (%)**	30.5±7.8	27.1±6.8	34.0±7.4	0.01*
**HGS (kgf)**	26.7±9.0	33.7±6.2	19.6±4.7	<0.001*
**6GST (m/s)**	1.4±0.4	1.5±0.34	1.3±0.4	0.07
**6MST (cycles)**	211.3±98.7	242.7±99.7	179.9±90.1	0.08
**Comorbidities**				
Cancer (%)	13 (43.3)	6 (40.0)	7 (46.7)	-
COPD (%)	4 (13.3)	2 (13.3)	2 (13.3)	-
Hypertension (%)	14 (46.7)	6 (40.0)	8 (53.3)	-
Diabetes Mellitus (%)	9 (30.0)	7 (46.7)	2 (13.3)	-
Dyslipidemia (%)	9 (30.0)	7 (46.7)	2 (13.3)	-
**Reason for hospitalization**				
Clinical	20 (66.7)	9 (60.0)	11 (73.3)	-
Surgical	10 (33.3)	6 (40.0)	4 (26.7)	-

kg: kilograms; m: meters; BMI: body mass index; MMSE: mini mental state examination; CCI: Charlson comorbidity index; HGS: handgrip strength; kgf: kilogram force; 6GST: six-meter gait speed test; s: seconds; 6MST: six-minute stepper test; COPD: chronic obstructive pulmonary disease;

*significant difference between men and women.

**Fig 2:**
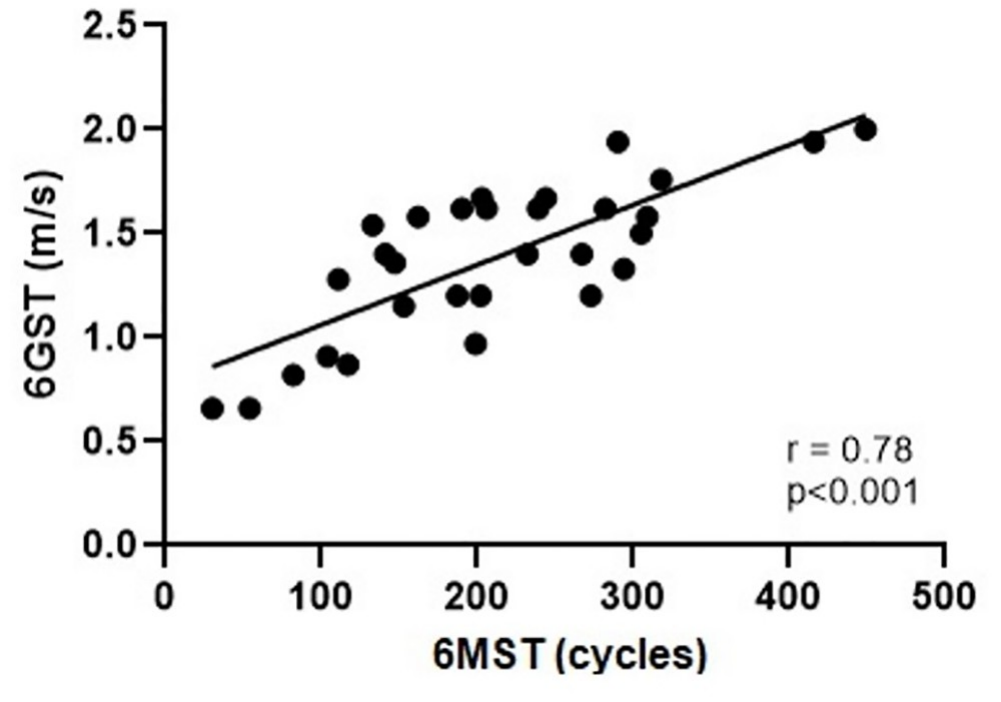
Correlation between performance in 6MST and 6GST. 6GST: six-meter gait speed test; 6MST: six-minute stepper test.

**Fig 3:**
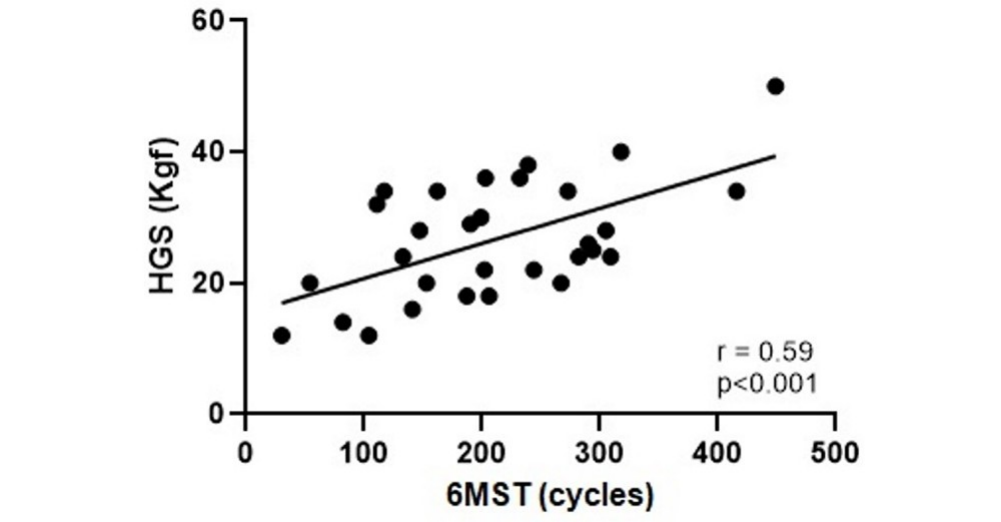
Correlation between performance in 6MST and HGS. HGS: handgrip strength; 6MST: six-minute stepper test.

**Fig 4:**
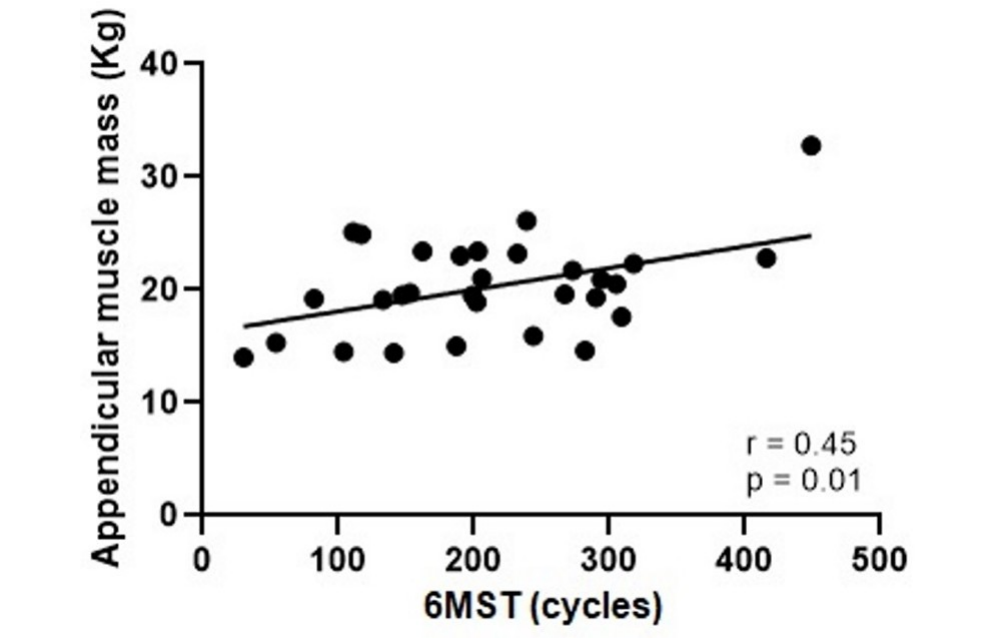
Correlation between performance in 6MST and appendicular muscle mass. 6MST: six-minute stepper test.

A high correlation was observed between 6MST and 6GST (r = 0.78; p<0.0001) and a moderate correlation between 6MST and HGS (r = 0.59; p<0.0001), Figs 2 and 3, respectively. With the appendicular muscle mass, the correlation was low (r = 0.45; p = 0.01), Fig 4. Thus, it was observed that the 6MST showed a correlation with all the parameters used for the diagnosis of sarcopenia, especially with variables related to functionality.

**Fig 5:**
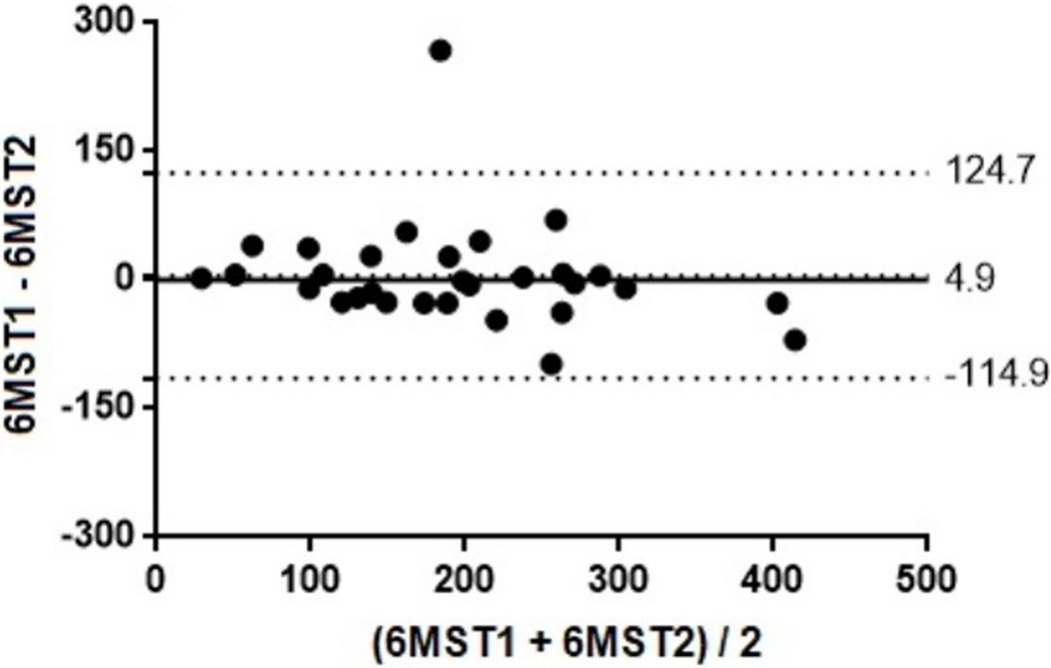
Bland-Altman plot of 6MST test-retest reliability in a hospital environment. 6MST: six-minute stepper test.

Regarding test-retest reliability, no statistical difference was observed between the average of the first and second 6MST performed by hospitalized elderly (196.2 ± 91.0 cycles vs. 191.3 ± 103.7 cycles; p = 0.66). In addition, there was an excellent agreement between these measures (ICC = 0.90; 95% CI 0.78–0.95). The Bland-Altman plot demonstrated this agreement (Fig 5).

**Fig 6:**
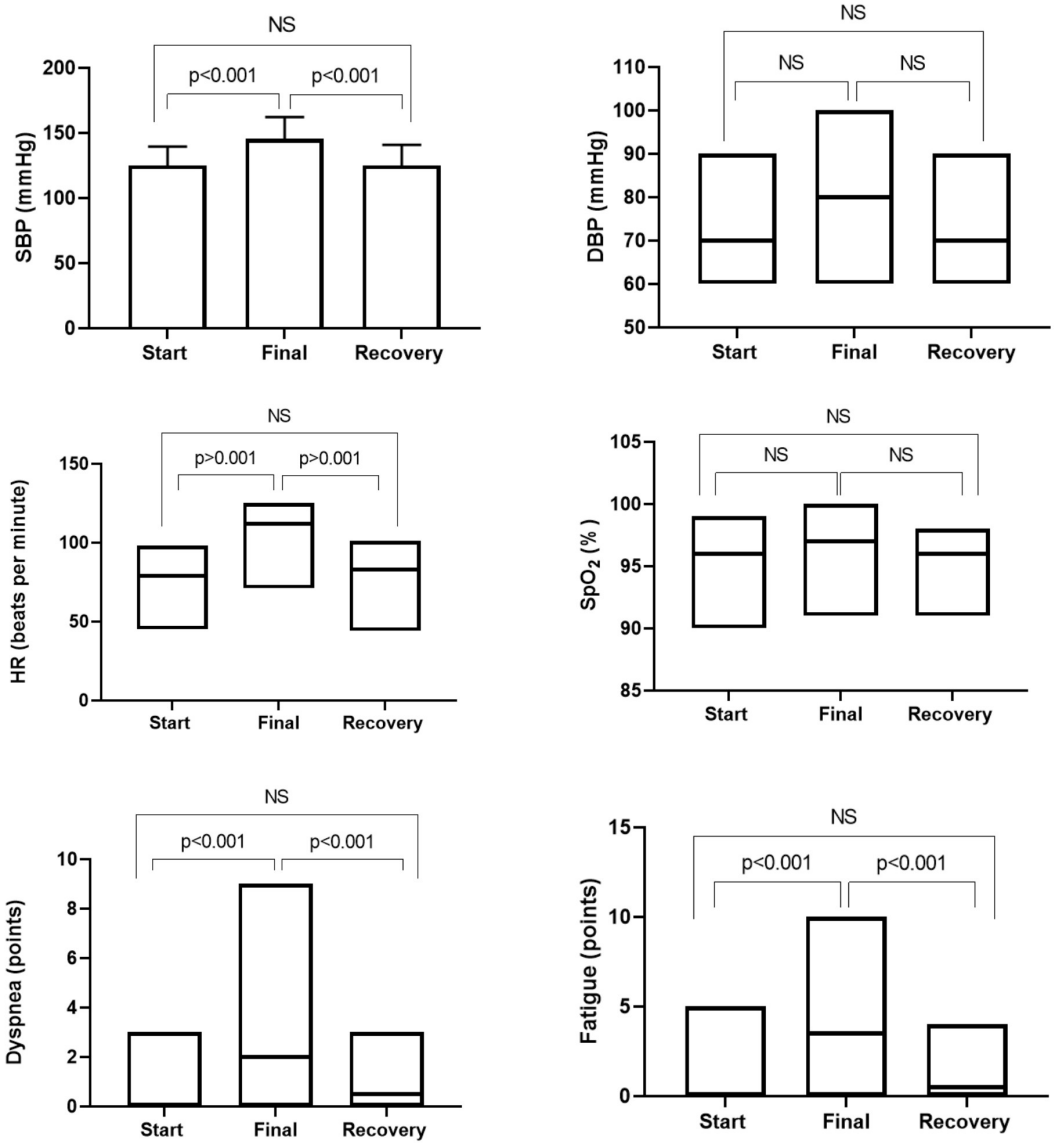
Behavior of vital signs and subjective sensation of dyspnea and fatigue of lower limbs of elderly patients hospitalized at the moments: Start, final and recovery of the 6MST. SBP: systolic blood pressure; DBP: diastolic blood pressure; HR: heart rate; SpO_2_: oxygen pulse saturation; NS: not significant.

Regarding safety, there was only one adverse event (3.3%) in the sample (MAP > 120mmHg at the end of the test), which did not generate clinical repercussions. Fig 6 shows the behavior of vital signs and the subjective sensation of dyspnea and fatigue of lower limbs at rest, at the end of the 6MST and in the recovery period six minutes after the end of the test. It was possible to observe that the values increased statistically at the end of 6MST. However, there was no statistical difference between the values at the start and after the recovery period of the 6MST.

**Fig 7:**
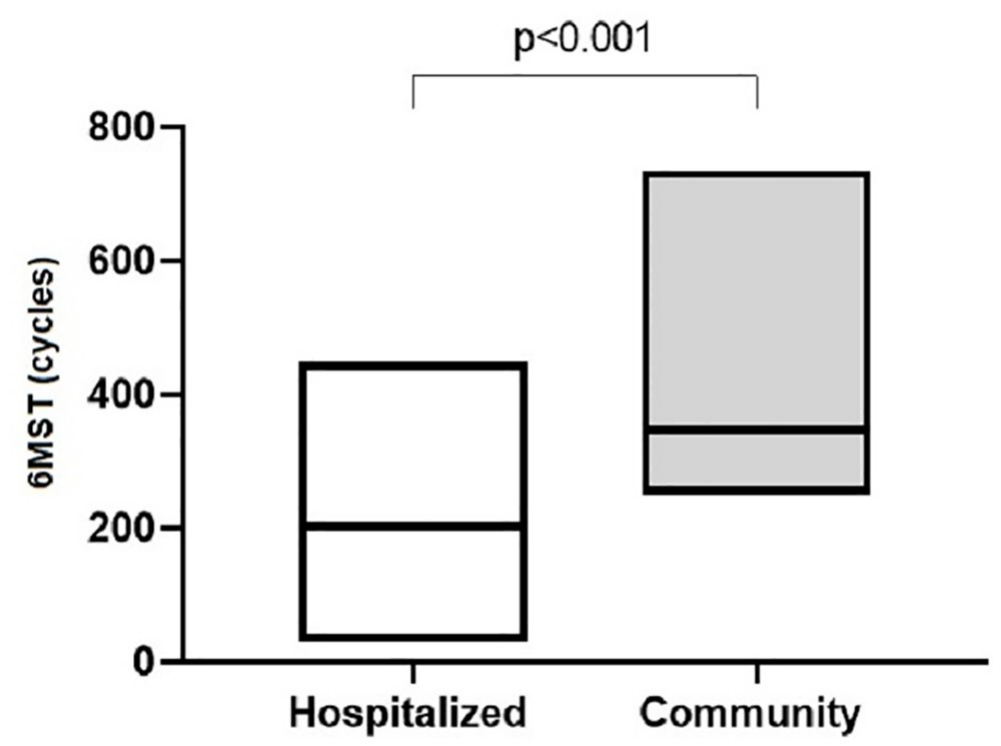
Comparison of performance in 6MST between hospitalized elderly and community elderly. 6MST: six-minute stepper test.

Table 2 reports the data of the hospitalized elderly and their healthy peers from the community. The Fig 7 shows the performance in 6MST, in absolute value, among hospitalized elderly and community elderly (p < 0.001).

**Table 2:** Comparison of variables between hospitalized elderly and community elderly.

**Variables**	**Hospitalized elderly (n = 23)**	**Community elderly (n = 15)**	**p**
**Age (years)**	68.1±5.9	68.1±5.8	1.00
**Weight (kg)**	69.8±14.0	68.1±11.5	0.66
**Height (m)**	1.7±0.9	1.6±0.8	0.10
**BMI (kg/m**^**2**^**)**	25.2±4.3	25.9±3.1	0.55
**6MST (cycles)**	203 (133–290)	346 (311–389)	<0.001*

kg: kilograms; m: meters; BMI: body mass index. 6MST: six-minute stepper test;

*significant difference between hospitalized elderly and community elderly.

## Discussion

The main result found in this study was that 6MST has convergent validity with the functional variables used in the diagnosis of sarcopenia, with a high correlation with 6GST and a moderate correlation with HGS. These results were expected, as studies have already indicated worse exercise capacity in individuals with sarcopenia, when compared to individuals without the dysfunction [5, 33, 34]. Possibly, the structural alterations of the skeletal muscle make it difficult to capture energy substrate and favor fatigue [5, 34]. Thus, patients with worse muscle function tend to have lower functional performance, and 6MST was able to demonstrate this agreement in an elderly hospitalized population.

Another important result observed in this study was an excellent reliability between the first and second 6MST when applied to hospitalized elderly. There was no statistical difference between the mean of the first and the second assessments. This information suggests the need for only one test to assess the exercise capacity of this population, which would facilitate its applicability in a hospital environment. Contrary to our findings, three previous studies observed a statistical difference between the first and the second 6MST, when used to assess elderly people in the community and patients with chronic obstructive pulmonary disease [15, 20, 35]. The authors justify that the best performance in the second test could be related to the learning effect or the hydraulic heating of the device’s piston. Therefore, future studies should investigate the test-retest reliability of 6MST when applied to hospitalized populations with specific chronic diseases, such as: cancer, liver cirrhosis, decompensated chronic heart failure or exacerbated chronic obstructive pulmonary disease.

In our sample of 30 patients, there was only one adverse event (3.3%), with an episode of hypertension (MAP > 120mmHg) at the end of the test. However, the patient remained asymptomatic and the pressure variables returned to normal levels in the recovery period after 6MST. Confirming the safety of the test, when comparing vital signs and the subjective sensation of dyspnea and fatigue of lower limbs, there was a statistical increase in almost all variables at the end of 6MST, but with a return to baseline after the period of recovery. This increase in vital signs during the evaluation was expected, as during exercise some acute cardiovascular changes occur to supply the metabolic demand. It is already established in the literature that during effort there is an increase in sympathetic activation, resulting in increased HR, myocardial contractility, stroke volume and SBP [36]. Therefore, 6MST is considered a safe tool for assessing the exercise capacity of hospitalized elderly patients. However, it is recommended that these individuals be monitored for at least six minutes after the end of the test, with a view to certifying the return of vital signs to baseline level.

It was observed that the performance of 6MST in hospitalized elderly was lower compared to that performed by their peers from the community. This finding can be justified by the presence of chronic diseases and the immobilization generated by hospitalization. In hospitalized elderly people, it was possible to observe the presence of cancer (43.3%) and chronic obstructive pulmonary disease (13.3%), which are comorbidities with a negative impact on exercise tolerance. The presence of dyspnea, altered lung function, systemic inflammation, malnutrition, reduced muscle function, fatigue, use of cardiotoxic therapies, and sedentary profile are possible factors that contribute to the reduction of exercise capacity [37–39]. The hospitalization period, on the other hand, can further favor immobility and, consequently, the reduction of muscle function and exercise tolerance [10], justifying this worse performance.

In the analysis between genders of hospitalized elderly group, there was a statistical difference in the anthropometric variables (weight and height), body composition (total muscle mass, appendicular muscle mass and body fat tissue) and HGS. These differences were expected, since in the literature lower values of appendicular muscle mass and HGS are reported for women [6]. However, in the two hospitalized groups, the mean values of these parameters were above the cutoff points defined by the European Working Group on Sarcopenia in Older People for the diagnosis of sarcopenia [6]. Regarding the performance in 6MST between hospitalized men and women, there was no statistical difference. However, when comparing the average value performed by the groups, it was observed that the cycles performed by men presented a difference of 62.8 cycles (26%) when compared to women. This difference is higher than the minimally important difference established for the 6MST (20 cycles) [40]. In addition, it can be suggested that our sample was small to reflect the statistical difference between these groups. Considering the values observed in our study, the sample needed to answer this question should be consisted of 41 hospitalized men and 41 hospitalized women.

In the present study patients were recruited in a non-probability method, for convenience. A limitation of using this kind of recruitment is the risk of not having a representative sample of the population. However, we can highlight that there was no dominant gender and that chronic diseases (hypertension, diabetes mellitus, chronic obstructive pulmonary disease and cancer) normally found in the hospitalized elderly were also found in our sample. The lack of control over variables that could influence the results of body composition, such as the hydration level, is another limitation of this study. However, water balance is not a measure performed in all patients, making this control difficult during research. As the secondary objective of our study was to compare the performance in the 6MST between hospitalized elderly and healthy elderly, only an anthropometric assessment was performed to match the subjects. A simpler evaluation in the healthy group happened to reduce the assessment time in order to improve adherence of the subjects in this group. In view of that, not carrying out other assessments in the healthy group can be considered as one of the limitations of our study, since we couldn´t compare mental status, muscle mass, muscle strength and gait speed between groups. However, the purpose of this study was to highlight the importance of monitoring exercise capacity in a hospital environment, especially in the elderly population, which has factors that lead to functional decline. For this purpose, 6MST seems to be a good tool, as it has convergent validity with other functional variables, excellent test-retest reliability and safety during its application in hospitalized elderly. Thus, the use of this tool should be encouraged by clinicians, aiming to screen hospitalized patients who must be included in in-hospital rehabilitation programs and after discharge. In addition, performance in 6MST could be used as an indicator of in-hospital quality, reflecting the impact of the rehabilitation service on the exercise capacity during the hospitalization period.

## Conclusion

The 6MST showed convergent validity with the functional variables used in the diagnosis of sarcopenia. In addition, excellent test-retest reliability was observed, which indicates the need for a single assessment in hospitalized elderly patients. The prevalence of adverse events during the application of the test is low, without resulting in clinical symptoms; therefore, the test can be considered safe for this population. In addition, hospitalized elderly patients perform worse in the 6MST compared to healthy elderly from the community.

## Supporting information

S1 Data (XLSX)
